# Major Outer Membrane Protein from *Legionella pneumophila* Inhibits Phagocytosis but Enhances Chemotaxis of RAW 264.7 Macrophages by Regulating the FOXO1/Coronin-1 Axis

**DOI:** 10.1155/2021/9409777

**Published:** 2021-11-13

**Authors:** Zehui Yang, Yingying Chen, Qiang Zhang, Xiaodong Chen, Ze Deng

**Affiliations:** ^1^Department of Pulmonary and Critical Care Medicine, Shengjing Hospital of China Medical University, Shenyang 110004, China; ^2^Department of Oncology, Shengjing Hospital of China Medical University, Shenyang, Liaoning 110000, China

## Abstract

*Legionella pneumophila* is an intracellular pathogen that can cause Legionnaire's disease by invading alveolar epithelial cells and macrophages. The major outer membrane protein (MOMP) plays an important role in the interaction between bacteria and host cells. However, the role of MOMP in the process of *L. pneumophila* invasion of macrophages and its working mechanism remain unknown. We aimed to explore the effects of MOMP on phagocytosis and chemotaxis of RAW 264.7 macrophages. The chemotactic activity, toxicity, and phagocytosis of RAW 264.7 cocultured with different concentrations of MOMP were determined by Transwell, CCK-8, and neutral red uptake assays, respectively. Target genes were detected by double-luciferase and pull down assays. qRT-PCR and Western blot were performed to analyze the expression of several important proteins involved in the immune response pathway, including coronin-1, interleukins (IL-10), forkhead transcription factor 1 (FOXO1), nucleotide-binding oligomerization domain protein (NOD) 1, NOD2, and receptor-interacting protein (RIP) 2. After coculturing with MOMP, cytological observation indicated a decrease of phagocytosis and a marked increase of chemotaxis in RAW 264.7 macrophages. The phagocytosis degree of RAW 264.7 macrophage varied with the concentration gradient of MOMP in a time-dependent manner. MOMP could increase the expression levels of MCP-1, IL-10, NOD2, and RIP2 and decrease the expression levels of FOXO1 and coronin-1 in cell culture supernatants. In addition, we found that FOXO1 could promote its transcription by binding to the promoter of coronin-1. The results of the present study suggested that MOMP could inhibit phagocytosis and facilitate chemotaxis of RAW 264.7 macrophage, which might be associated with the FOXO1/coronin-1 axis.

## 1. Introduction

*L. pneumophila*, a Gram-negative bacterium of the legionella genus, has gained considerable attention for the causing pneumonia (known as Legionnaires' disease) outbreak since 1976 during the veterans' convention in Philadelphia, United States [1, 2]. This pathogenic bacterium is widely distributed in nature and easy to be spread and transmitted in aerosols produced by various natural water sources, artificial cooling, or hot water piping systems, such as hospital air conditioning, cooling water, showerheads, and auxiliary ventilators. When exposed to the aerosols contaminated by *L. pneumophila*, individuals can be infected almost exclusively by aerosol inhalation, allowing bacteria juxtaposed with alveolar macrophages [1].

The pathogenicity of *L. pneumophila mainly* stems from the following two factors. The first is the surface structure of the bacteria which is dependent on flagella, pili, and membrane proteins [3]. According to different adhesion mechanisms, the pathogenic process of *L. pneumophila* can be divided into complement- and non-complement-dependent adhesion. The complement components C3 and C3bi are the key proteins that belong to the complement system in the former adhesion process, which are primarily fixed on MOMP encoded by cloning and the nucleotide sequence of a gene (ompS) on the *L. pneumophila* surface [4]. They subsequently bind to the macrophage complement receptors CR1 and CR3 on the surface of target cells [5]. Meanwhile, flagella, pili, and surface proteins also interact with target cells through macrophage infectivity potentiator (MIP) encoded by the MIP gene, heat shock protein 60 encoded by htB, type IV pilin encoded by pilB/C/D, and the flagellin encoded by flaA to achieve the non-complement-dependent adhesion [6–8]. The other pathogenic factor of this bacteria is attributable to the pathogenicity island (PAI) associated with the secretory system. Many PAIs are discovered in *L. pneumophila* with different etiological characteristics including Cpx, dot/icm complex, lvgA PAI (virulence protein), and irAB which belongs to −65 kb PAI. The intracellular and extracellular tolerance mechanisms of the PAI function is essential for causing intracellular infections [9–11].

As a type of antigen-presenting cell, macrophages are widespread and associated with immunity, homeostasis, and wound healing in fundamental functions. They also play a vital role in identifying and clearing cell apoptosis. A remarkable feature of this process is the absence of inflammation [12]. *L. pneumophila* enters macrophages by heterogeneous pathways and changes vacuole maturation in a way that is conducive to their survival [13]. The MOMP of *L. pneumophila* binds complement components C3 and C3bi and mediates bacteria uptake via the complement receptors CR1 and CR3 of macrophages. Some studies have shown that MOMP can potentially mediate human macrophages to phagocytize *L. pneumophila* [4, 14], but how it affects macrophages has not been elucidated. The present study detected the chemotactic function of macrophages using Transwell and the phagocytosis function of macrophages using neutral red phagocytosis following the treatment of MOMP on RAW264.7 macrophages. When *L. pneumophila* infects alveolar macrophages, it can cause a series of immune response to affect the biological function of macrophages. It is published before the NOD1 and NOD2 and their adaptor serine-threonine kinase RICK: kinase (Rip2) were related to immune responses induced by bacterial infection [15]. Furthermore, FOXO1 and coronin-1 play a role in neutrophil function and phagosome maturation. Therefore, analyses were made to determine whether MOMP affected immune response factors and function of macrophages including IL-10 [16], Rip2, NOD1, and NOD2 [17].

## 2. Materials and Methods

### 2.1. Cell Line and Growth Condition

RAW 264.7 macrophage cell lines were obtained from Biomedicine Biotech (Chongqing, China) and maintained in Dulbecco's modified Eagle's medium (Solarbio Science & Technology, Beijing, China) with 10% fetal bovine serum and cultured in a humidified incubator with 5% CO_2_ at 37°C.

### 2.2. Plasmid

The recombinant plasmid pET-MOMP of *L. pneumophila* was constructed by our research team and stored at −20°C [18, 19]. The plasmids of backbone vectors pGL3-basic, pRL-TK, and pcDNA3.1(+) were bought from an agent of Promega (Beijing, China).

### 2.3. Antibody

Antibodies for IL-10 (BM12255), NOD1 (BM1246), NOD2 (BM15992), MCP-1 (BM7277), coronin-1 (BM9300), FOXO1 (BM2934), anti-RIP2 (BM2498), and *β*-actin (BMC038) were obtained from Biomedicine Biotech (Chongqing, China).

### 2.4. Cell Counting Kit-8 (CCK-8) Assay

The toxicity of MOMP to RAW 264.7 macrophages and 50% inhibitory concentration (IC50) were detected by the CCK-8 assay. Cells in the logarithmic phase of growth were neutralized by conventional digestion and resuspended evenly using fresh culture mediums, and the relative cell activity was calculated finally. The concentrations of cells were adjusted to 6 × 10^4^ cells/ml. The cell suspension was added to 96-well plates, 100 *μ*l for each well (approximate 6 × 10^3^ cells/well), and incubated overnight at 37°C with 5% CO_2_. The purified MOMP (20 *μ*g/ml) was diluted with a complete medium at 1 : 2, 1 : 4, 1 : 8, 1 : 16, 1 : 32, and 1 : 64 ratios; the solution was subsequently transferred to the plates, 100 *μ*l per well. Meanwhile, blank (the culture medium without cells adding CCK-8) and control (the culture medium seeding cells without CCK-8) wells were set with three multiple wells for each group. Following treatment with different concentrations of MOMP for 24 h, 20 *μ*l of CCK-8 reagent (Engreen Biosystem, Beijing, China) was added to each well and incubated for 3 h at 37°C. The optical density (OD) was measured by a microplate reader (Beckman Coulter, Fullerton, CA) at 450 nm. Each concentration was detected in triplicate in the 96-well plates, and the experiments were repeated independently at least three times. The IC50 was calculated using GraphPad Prism software (version 8.0.2). According to the value of IC50, three concentrations of MOMP were set for further experiments including a low-dose group (0.05 × IC50), a medium-dose group (0.1 × IC50), and a high-dose group (0.2 × IC50).

### 2.5. Neutral Red Uptake Assay

Neutral red uptake was performed to measure the effects of MOMP on the phagocytic activity of RAW 264.7 cells. 100 *μ*l freshly suspended RAW 264.7 cells were seeded into 96-well plates (approximately 5 × 10^4^ cells/well) and incubated overnight at 37°C with 5% CO_2_. Mediums containing different concentrations of MOMP were added into the cell suspension (100 *μ*l per well). The wells containing 100 *μ*l medium were taken as the control group and 5 repetitions were made for each group. Following incubation at 37°C with 5% CO_2_ for 24, 48, and 72 h, 100 *μ*l of neutral red staining solution (Solarbio, Beijing, China) preheated at 37°C was added to each well and the medium was discarded and washed with PBS twice. Following 2 h of incubation at 37°C, the neutral red medium was removed and the cells were washed with 150 *μ*l PBS per well by covering or immersing the plates in PBS. The washing solution was removed by gentle tapping three times. 150 *μ*l neutral red destaining solution (50% ethanol, 1% glacial acetic acid, and 49% deionized water) was added in each well, and the plate was shaken rapidly on a plate shaker for 10 min to fully suspend the cells. Finally, the OD value at 540 nm was measured by a microplate reader, using the blank containing no cells as a reference. The macroscopic changes in the neutral red uptake condition of the cells were recorded through a phase-contrast inverted microscope. The neutral red uptake assay was designed and performed as the protocol reported by Repetto et al. [2].

### 2.6. Transwell Assay

Comparative chemotaxis of RAW 264.7 macrophages was conducted using a conventional 24-well Transwell system (Corning, NY, USA) after being treated with MOMP. A microporous polycarbonate membrane (with 8 *μ*m of pore size and 10 *μ*m of thickness) was used for isolating the upper chamber from the lower well. The cells were seeded in the chamber; when the chamber was placed in the 24-well plate, the macrophages were able to transfer from the chamber to the lower well through the polycarbonate membrane. Detailed procedures were described as follows: the cell density of RAW264.7 was initially adjusted into 5 × 10^5^ cells per ml medium. 100 *μ*l of cell suspension was inoculated into the upper chamber, and 500 *μ*l of DMEM containing different concentrations of MOMP was added to the lower well. The lower well was taken as the control where the medium had just been added, with six repeats for each well. The chamber was placed on a bubble-free plate and incubated at 37°C with 5% CO_2_. 24 h later, the upper chamber was removed and washed twice with PBS. The cells that remained in the chamber for not passing through the membrane were gently transferred to the well containing 500 *μ*l of 4% paraformaldehyde and left undisturbed for 30 min. After fixation, the chamber was turned upside down on a piece of absorbent paper and dried naturally. And the chamber was subsequently stained in the dark being placed into a well containing 0.1% crystal violet (Solarbio, Beijing, China) for 15 min. To measure the chemotaxis index, the crystal violet and nonadherent cells were then rinsed away from the chamber with ddH_2_O and pictures of adherent cells were photographed under a microscope when the chamber was dry. The chemotaxis index was defined as follows: the number of cells of treatment group/number of cells in control, calculated by eluting crystal violet with a 33% acetic acid solution. The OD value of the eluent was measured at 570 nm. The formula calculated was presented as follows: chemotaxis index (%) = (OD of treatment/OD of control) × 100%. The medium treated with different doses of MOMP in the lower well was centrifuged at 3000 rpm for 10 min, and the supernatant was collected for ELISA assay using mouse MCP-1 and IL-10 ELISA Kit (Elabscience, Wuhan, China) according to the manufacturer's instructions.

### 2.7. Total RNA Isolation and qRT-PCR

The cells were treated with different concentrations of MOMP for 24 h and cultured in a MOMP-free medium containing different concentrations of MOMP, and expression levels of genes related to phagocytosis and chemotaxis were determined. Total RNA was extracted from the cells using RNAiso plus (TaKaRa, Dalian, China) according to the manufacturer's instructions. 2 *μ*g of total RNA from each sample was subjected to qRT-PCR using PrimeScript™ RT reagent kit (Takara, Dalian, China) on Applied Biosystems 7300 Real-Time PCR System (Applied Biosystems, Foster City, CA) following the recommended procedures. Primers used for real-time PCR were listed in Table 1, and the PCR program was set as follows: one cycle of 95°C for 10 s was followed by 40 cycles of 95°C for 5 s and 60°C for 30 s. The procedures were repeated three times for the control. The average *Ct* value of each group was taken to calculate the relative expression level using the method of 2^−△△*Ct*^, where ^△^*Ct* = *Ct* (target gene) − Ct (action gene) and ^△△^*Ct* = ^△^*Ct* (treatment) −  ^△^*Ct* (control).

### 2.8. Western Blotting

The cells and culture supernatant treated with MOMP in different concentrations were collected, and Western blotting was performed for further analysis. The total protein was extracted with TCA-acetone (trichloroacetic acid-acetone) and denatured completely for later use. The proteins were transferred to a polyvinylidene fluoride (PVDF) membrane being separated with 10% SDS-PAGE. Then, the immunostaining was performed in the sequences of blocking, the primary antibody incubation, membrane balancing and washing, secondary antibody incubation, and reaction termination by membrane washing. The primary antibodies were diluted with QuickBlock™ primary antibody dilution buffer (Beyotime, Shanghai, China) of IL-10 and FOXO1 at a 1 : 1000 dilution ratio; 1 : 1500 to NOD1 and coronin-1; 1 : 2000 to RIP2, NOD2, and MCP-1; and 1 : 2500 to *β*-actin used as an internal control. The secondary antibodies labeled with horseradish peroxidase were diluted with QuickBlock™ secondary antibody dilution buffer at a ratio of 1 : 5000. PVDF membranes were exposed by ECL-enhanced chemiluminescence and the results were observed on a PVDF film imager. The protein content was measured according to the grey value analyzed by the ImageJ software. Relative protein expression was quantified by the ratio of gray values of target protein and internal control *β*-actin.

### 2.9. DNA Pull Down Assay

DNA pull down assay was performed to find out protein interaction with coronin-1. The 5′-flanking fragment of coronin-1 was labeled with biotin by PCR amplification using 5′-terminal biotinylated forward primers (Table 1). The gene was bound to streptavidin magnetic beads using the Dynabeads KilobaseBINDER Kit following the manufacturer's protocol (Invitrogen). The nuclear proteins were incubated with biotinylated DNA—Dynabeads complex on a rotating shaker at 4°C overnight. The supernatant was removed and the complex was washed three times with cold PBS following the incubation. After the last washing, a pull down solution was resuspended with eluent at 70°C for 3 min to break the binding of streptavidin and biotin. The eluted DNA protein from the beads that did not contain the biotinylated DNA probe was used as the control, and Western blotting was performed to identify the specific protein.

### 2.10. Dual-Luciferase Reporter Assay

To investigate the interaction between the transcription factor FOXO1 and the coronin-1 promoter, two expression vectors containing firefly luciferase and Renilla luciferase were employed to perform the dual-luciferase reporter assay. The following four plasmids were prepared including pGL3-coronin1-pro-luc+, pcDNA3.1(+)-FOXO1, pGL3-basic-luc+ (Promega), and pRL-TK-Rluc (Promega). The primers for fragment cloning of FOXO1 and coronin-1 were listed in Table 1. The pGL3-basic was the backbone of pGL3-coronin1-pro and it was set as the negative control. pRL-TK was intended to be used as an internal control reporter. Using TransFast™ Transfection Regent (Promega E2431), the prepared RAW 264.7 cells were divided into three groups for transfection. The cells of group 1 were cotransfected with pGL3-coronin1-pro-luc+, pcDNA3.1(+)-FOXO1, and pRL-TK-Rluc, with a ratio of 3 : 3 : 1. The cells of group 2 were cotransfected with pGL3-coronin1-pro-luc+ and pRL-TK-Rluc with a ratio of 3 : 1, and the cells of group 3 were with pGL3-basic-luc+ and pRL-TK-Rluc at 3 : 1 ratio. More detailed information about vector recombination and transfection protocol is available on the website at http://www.promega.com/tbs/. Following 24 h transfection, the cells were harvested and the firefly and Renilla luciferase activities were measured using a Dual-Luciferase® Reporter Assay System (Promega) according to the manufacturer's instructions. All the data were obtained from three parallel experiments and the average values were taken for further analysis. The activities of firefly luciferase were subsequently normalized by internal control and analyzed using GraphPad Prism software (version 8.0.2).

### 2.11. Statistical Analysis

All data were analyzed with SPSS 23.0 software (SPSS, USA) and indicated in mean values with ±SD. One-way ANOVA (post hoc test: Tukey's multiple comparison test) or a Student's *t*-test was used to examine statistical significance through comparison of mean values in different groups. *P* values were used to determine the significance, and *P* < 0.05 was considered statistically significant. The statistical significance was labeled by an alphabetic notation as well (a, b, c, and d), to mark the significant differences in the figure legend.

## 3. Results

### 3.1. MOMP Inhibits the Proliferation and Phagocytic Activity of RAW 264.7

**Figure 1:**
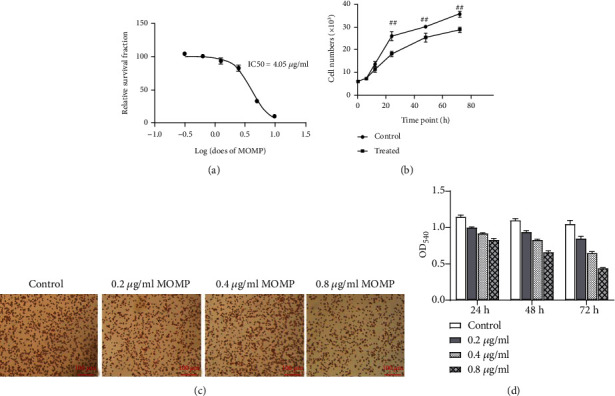
MOMP inhibits the proliferation and phagocytic activity of RAW264.7. (a) A dose-response study of MOMP indicated IC50 as 4.05 *μ*g/ml. The curve was fitted using relative survival fraction of six working concentrations (10, 5, 2.5, 1.25, 0.625, and 0.3125 *μ*g/ml). (b) The proliferating capacity of RAW 264.7 upon the treatment of MOMP. Statistical analysis by one-way ANOVA, ^##^*P* < 0.01. Error bars = 95% confidence intervals. (c) The cytological phagocytosis of RAW 264.7 by neutral red uptake assay at 24 h in the four groups (control; cells treated with 0.2 *μ*g/ml MOMP; cells treated with 0.4 *μ*g/ml MOMP; and cells treated with 0.8 *μ*g/ml MOMP) (original magnification: 200x, scale bar = 100 *μ*m), *P* < 0.05 indicated significant differences in neutral red uptake of cells between control and treated groups. (d) Semiquantitative analysis of the phagocytosis of RAW 264.7. The significant differences were marked by the alphabetic notation (a, b, c, and d). In each same time point of different concentration treatment groups, if any two histograms with the same marking letter represented there have no significant difference between two concentration treatments, any two histograms without the same marking letter represented there have a significant difference.

As MOMP is considered to serve as an important virulence factor of *L. pneumophila* [4], the toxicity of MOMP to RAW 264.7 macrophages was detected by the CCK-8 assay. The relative survival of macrophages was measured in the presence of decreasing concentrations of MOMP (10, 5, 2.5, 1.25, 0.625, and 0.3125 *μ*g/ml), and the IC50 curves were fitted using log (MOMP) versus relative survival fraction: variable slope (four parameters). Here, IC50 which was 4.05 *μ*g/ml was obtained. It was used to set the appropriate MOMP of different working concentrations. Based on the IC50 value, three concentrations of MOMP were set for the experiments: a low-dose group (0.05 × IC50 = 0.2 *μ*g/ml), a medium-dose group (0.1 × IC50 = 0.4 *μ*g/ml), and a high-dose group (0.2 × IC50 = 0.8 *μ*g/ml). Meanwhile, the cell number was calculated overtime at 4.05 *μ*g/ml MOMP. Compared with untreated cells, the treated showed marked toxic changes and elevated as MOMP concentration increased (Figure 1(a)) and the passage of time (Figure 1(b)). The mechanism of neutral red uptake assay was based on the ability of viable cells to incorporate the supravital dye neutral red in the lysosomes, so it was performed to measure the effects of MOMP on the phagocytic activity of RAW 264.7 cells. Following 24 h treatment with MOMP, the phagocytosis ability of RAW 264.7 macrophages treated with different doses of MOMP was lower than that of the control group (*P* < 0.05) (Figure 1(c)). The phagocytic activity could be determined by detecting the OD540 or visual stained cells in red under a microscope, for the macrophages would engulf the neutral red dye, and thus, the cells appeared in red. Therefore, as shown in Figure 1(c), the phagocytic activity was receded with the increase of MOMP concentrations. Semiquantitative analysis of the RAW 264.7 phagocytosis of neutral red showed that phagocytosis of cells treated with the same concentration of MOMP decreased over time compared with the control group (*P* < 0.05). Meanwhile, the RAW 264.7 macrophage phagocytosis of neutral red decreased with the increase of MOMP protein concentrations in a time-dependent manner (Figure 1(d)).

### 3.2. MOMP Enhances the Chemotaxis of RAW 264.7

**Figure 2:**
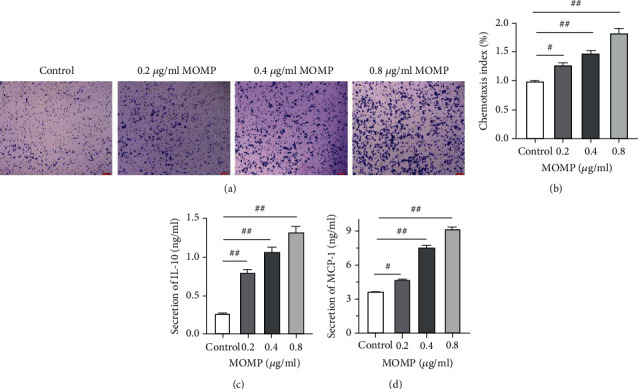
MOMP enhances the chemotaxis of RAW264.7 (a) The chemotactic potential of RAW 264.7 cells that migrated across the membrane in the MOMP-free medium, 0.2, 0.4, and 0.8 *μ*g/ml MOMP (crystal violet staining, original magnification: 200x, scale bar = 100 *μ*m). (b) Chemotaxis index of RAW 264.7. (c) Secretion level of IL-10 after the treatment of different doses of MOMP for 24 h. (d) Secretion level of MCP-1 after the treatment of different doses of MOMP for 24 h (*P* < 0.05, error bars = 95% CIs.). The significant differences were marked by a pound sign. *P* values: ^#^*P* < 0.05, ^##^*P* < 0.01.

The chemotaxis index results of macrophages have shown that the MOMP plays an important role in pathogen invasion [20]. The chemotaxis of RAW264.7 macrophages treated by different doses of MOMP was compared using a Transwell system. After 24 h, the cells were stained with crystal violet and observed under an optical microscope. Changes in cell chemotaxis were shown in Figure 2(a). Cytological observation revealed that cell count in the visual field of the culture medium treated with MOMP increased in the experimental range and the number of cells transferred from the upper chamber to the lower well increased with the increase of MOMP concentrations compared with that of the control group. Furthermore, the elevation of cell numbers was revealed in the visual field as the concentration of MOMP increased. In chemotaxis index analysis, the number of migrating cells was determined by measuring OD570 in control and treated conditions and the percentage of cells in the treated group compared to that in the control group was considered as the chemotactic ability of the cells. The higher the chemotaxis index, the higher the number of cells migrating towards the source of the chemotactic agent. The results indicated that the chemotactic activity of RAW264.7 macrophages increased in a dose-dependent manner treated with MOMP (Figure 2(b)**)**.

### 3.3. RAW 264.7 Secretes More IL-10 and MCP-1 in the Supernatant of Cell Culture Treated with MOMP

IL-10 is a cytokine produced by macrophages and it can inhibit the phagocytosis of macrophages [21]. MCP-1 is thought to have specific chemotactic activation [22]. To explore how MOMP affects the secretion of corresponding cytokines simultaneously inhibiting phagocytosis and enhancing chemotaxis, the contents of MCP-1 and IL-10 in the cell culture supernatant were detected by ELISA. The results (Figures 2(c) and 2(d)**)** showed that RAW 264.7 secreted more IL-10 and MCP-1 to the cell culture supernatant of MOMP-treated cells, which was consistent with the changing trend of phagocytosis and chemotaxis.

### 3.4. MOMP Increases NOD2 and RIP2 mRNA and Protein Levels and Decreases FOXO1 and Coronin-1 in RAW 264.7 Cells

**Figure 3:**
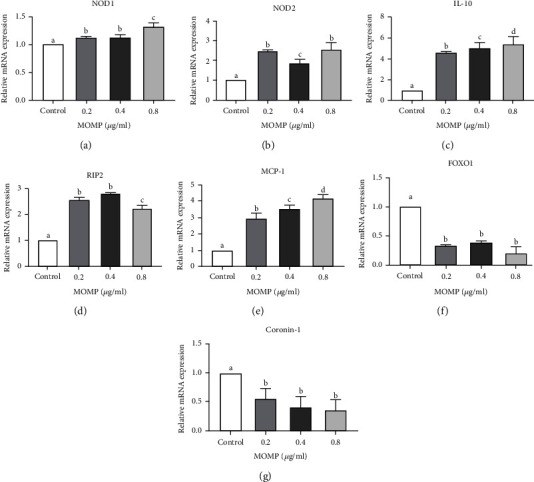
Analysis of mRNA expression of 7 proteins in RAW 264.7 treated with MOMP. The significant differences in the expression of 7 proteins in groups treated with different concentrations were marked by the alphabetic notation (a, b, c, and d). In each detected expression of 7 proteins, if any two histograms with the same marking letter represented no significant difference between two concentrations, any two histograms without the same marking letter represented a significant difference. (a–g) Relative mRNA expression analysis of NOD1, NOD2, IL-10, RIP, MCP-1, FOXO1, and coronin-1 for 24 h treated by different concentrations of MOMP. Control represented the cells cultured in a normal medium. 0.2 represented the cells cultured in the medium containing 0.2 *μ*g/ml MOMP, 0.4 represented the cells cultured in the medium containing 0.4 *μ*g/ml MOMP, and 0.8 represented the cells cultured in the medium containing 0.8 *μ*g/ml MOMP (error bars = SD).

**Figure 4:**
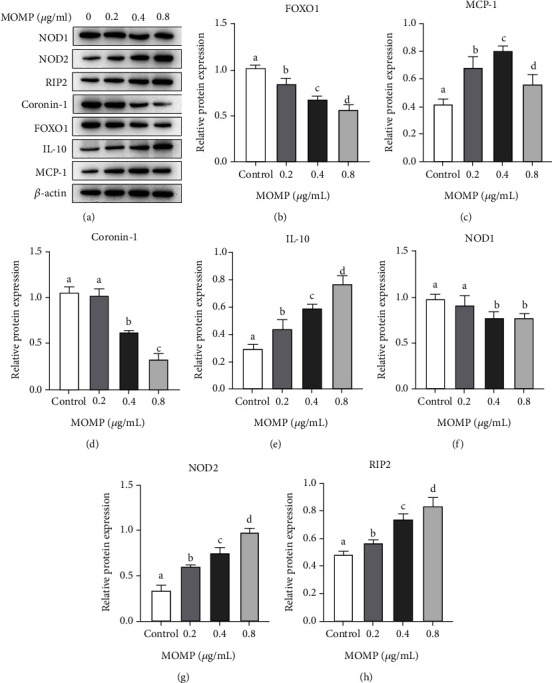
Expression analysis of 7 proteins at the protein level in RAW 264.7 treated by MOMP for 24 h. The significant differences in the expression of 7 proteins in groups treated with different concentrations were marked by the alphabetic notation (a, b, c, and d). In each detected expression of 7 proteins, if any two histograms with the same marking letter represented no significant difference between two concentration treatments, any two histograms without the same marking letter represented a significant difference. Relative protein expression analysis in each group was performed on the basis of the gray value of internal control *β*-actin. (a) Western blotting analysis of 7 proteins and *β*-actin. (b–h) Relative protein expression of FOXO1, MCP-1, coronin-1, IL-10, NOD1, NOD2, and RIP2 for 24 h treated by different concentrations of MOMP. Control represented the cells cultured in the normal medium; 0.2 represented the cells cultured in the medium containing 0.2 *μ*g/ml MOMP; 0.4 represented the cells cultured in the medium containing 0.4 *μ*g/ml MOMP; 0.8 represented the cells cultured in the medium containing 0.8 *μ*g/ml MOMP (error bars = SD).

To better understand the molecular mechanism of MOMP on phagocytosis and chemotaxis, a further analysis was conducted on the expression of proteins, which were reported to be involved in several kinds of immune response at the mRNA level in RAW 264.7 cells treated with different doses of MOMP for 24 h [23]. Meanwhile, the protein levels in RAW 264.7 cells treated with different doses of MOMP for 24 h were also analyzed. As reported before, IL-10 and RIP2 could inhibit the phagocytosis of macrophages [21, 24]; MCP-1 has a specific chemotactic activation effect on macrophages [22]; NOD1 and NOD1 expression were related to migration capacity, phagocytosis, and bacterial killing [25]; FOXO1 regulates neutrophil function that facilitates chemotaxis, phagocytosis, and bacterial killing [26], and coronin-1, an actin-binding protein, is involved in phagosome maturation. Therefore, IL-10 and MCP-1 and five additional proteins NOD1, NOD2, RIP2, FOXO1, and coronin-1 were selected as candidates in the present study. Overall, compared with untreated cells, all proteins showed statistically significant changes in mRNA (Figure 3) and protein levels (Figure 4) treated with different concentrations of MOMP. The mRNA expression of NOD2, RIP2, IL-10, and MCP-1 was upregulated and increased with the increase of concentrations (Figures 3(b)–3(e)) whereas FOXO1 and coronin-1 showed the opposite results in RAW 264.7 cells treated by MOMP (Figures 3(f) and 3(g)). The results of protein expression analysis were consistent with that of the analysis of mRNA expression (Figures 4(a)–4(h)).

### 3.5. Transcription Factor FOXO1 Binds Specifically to the Promoter Region of Coronin-1

**Figure 5:**
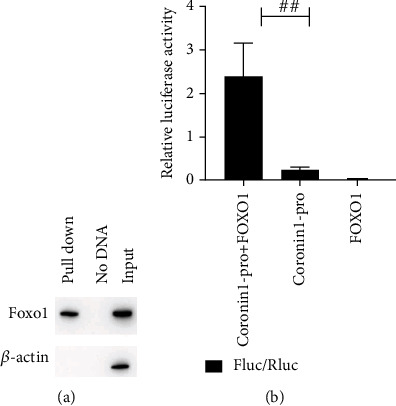
The transcription factor FOXO1 binds specifically to the promoter region of coronin-1. (a) Western blotting analysis results of DNA pull down assay. (b) Relative luciferase activity of cotransfection of different plasmids. Coronin1-pro+FOXO1 represented cotransfection of pGL3-coronin1-pro-luc+ and pcDNA3.1(+)-FOXO1; coronin1-pro represented the cells just transfected by pGL3-coronin1-pro-luc; FOXO1 represented the cells just transfected by pcDNA3.1(+)-FOXO1. The significant differences between relative luciferase activity of coronin1-pro+FOXO1 and coronin1-pro were marked by the pound sign. *P* values: ^##^*P* < 0.01.

Through expression analysis, both FOXO1 and coronin-1 were downregulated after MOMP treatment of RAW 264.7 cells. The result suggested that the two proteins might be related to phagocytosis and chemotaxis of RAW 264.7 macrophage by MOMP. Furthermore, FOXO1 serves as a transcription factor that may be involved in the negative regulation of cell proliferation, while coronin-1 is a protein that participates in the regulation of macrophage phagocytosis [27, 28]. We therefore performed a putative experiment to evaluate the interaction between the DNA-binding domain of FOXO1 and the coronin-1 promoter. The nuclear proteins that could interact with coronin-1 were pulled down by incubating with biotinylated DNA—Dynabeads complex, and the expression was detected by Western blotting; then, the dual-luciferase activity would increase when coronin-1 bound to FOXO1. The pull down assay and dual-luciferase reporter assay indicated that the Foxo1 in nuclear proteins could interact with coronin-1 and FOXO1 as a transcriptional regulator could affect the promoter activity of coronin-1 (Figures 5(a) and 5(b)).

## 4. Discussion

Legionnaires' disease is severe because legionella can invade alveolar macrophages and survive in the cells for a long time [29]. MOMP is considered a virulence factor that can promote bacteria to invade cells so it is used as a target for the identification of virulence factors and vaccines. The virulence factors include lipooligosaccharide, immunoglobulin-binding proteins, MOMP, other outer membrane proteins, and exopolysaccharide [30]. However, the role in the pathogenesis of *L pneumophila* remains unknown. The present study provides evidence that MOMP plays a critical role in the process of RAW 264.7 response to the *L. pneumophila* infection. Our data demonstrated MOMP could inhibit the phagocytosis and proliferation of macrophages in a time- and dose-dependent manner, which could contribute to *L. pneumophila* phagocytosis and avoid the interference of the immune system and the infection of alveolar cells. Meanwhile, the chemotaxis index analysis indicated the increased chemotactic activity of RAW 264.7 followed by treating with increased concentrations of MOMP. The secretion levels of IL-10 and MCP-1 in cells were determined, and the results showed that the secretion level increased with the increased concentrations of MOMP which were consistent with the decreased phagocytosis and the enhanced chemotaxis of RAW 264.7.

To further study the mechanism of MOMP activity on RAW 264.7. We also observed induced protein and mRNA production such as NOD1, IL-10, MCP-1, NOD2, and RIP2 in RAW 264.7 cells under MOMP stimulation compared to normal cells, which are thought to be involved in various immune responses against bacterial infection [31]. The results indicated the ability of MOMP in modulating the expression of proteins in cells to inhibit phagocytosis and enhance chemotaxis of RAW 264.7. MCP-1 [32], coronin-1 (an actin-associated protein) [33], FOXO1 [34], and RIP2 [1] are considered important in pathways of pathological injury and immune signal transduction. Meanwhile, the results following an investigation of these proteins on their regulatory role of MOMP showed that MOMP could increase the expression levels of MCP-1, IL-10, NOD2, and RIP2 in the cell culture supernatant and decrease the expression levels of FOXO1 and coronin-1 which was related to the phagosome maturation. The further DNA pull down and dual-luciferase reporter assay showed that FOXO1 could bind to the coronin-1 promoter and promote its transcription.

Coronin-1 is an actin-associated protein [35], which requires an early intervention in phagosome formation conforming to actin polymerization [36, 37]. In the absence of coronin-1 in phagocytes, the chemotaxis gathered in coronin-1 on the phagocyte membrane, activating the calcineurin signaling pathway and inhibiting the fusion of phagocytes and lysosomes, which is the mechanism of pathogens to evade immune killing. The significance of the coronin-1 signaling pathway is that it not only can lead to homologous immunity but also can avoid the infection of pathogenic microorganisms. On the basis of data analysis, we have observed that the expression of FOXO1 and coronin-1 was both downregulated in RAW 264.7 treated with MOMP. The results of this study suggested that MOMP could inhibit the phagocytic function of RAW 264.7 macrophages and enhance its chemotactic function, which might be associated with the FOXO1/coronin-1 axis. As *L. pneumophila* threatens the health of mankind, the present study is expected to provide a novel topic for the prevention and treatment of Legionnaires' disease.

## 5. Conclusions

MOMP played a critical role in affecting the response of RAW 264.7 to *L. pneumophila* infection. This process might be achieved by the synergistic regulation of FOXO1 and coronin-1. Meanwhile, FOXO1 could regulate the promoter activity of coronin-1 and the FOXO1/coronin-1 axis could be used as a novel candidate signaling pathway for the treatment of Legionnaires' disease.

## Figures and Tables

**Table 1:** Primers used in this study.

Primers used in qPCR analysis		
Gene	Forward primer (5′-3′ end)	Reverse primer (5′-3′ end)
Coronin-1	CTGAGGAATGGCTGGGTGGT	CTGGTGTAGCTCTTCTGCGA
IL-10	GAGACTTGCTCTTGCACTACC	TGCAGTTATTGTCTTCCCGC
FOXO1	CTGTGACATGGAGTCCATCA	TGTAGCCTGCTCACTAACTC
MCP-1	TACAGGAGTCTGTGATGATG	TGATGGCACTTCTCTTGCCT
NOD1	GTCAGTCTCAGAGGATCTCT	GTATGGTGGTGGGTATGTGC
NOD2	ATCCCCACGCTGCAGCTG	CATAGTCCTCCCTGGAGAG
RIP2	TGCCTCCTGTCTGTCTCCAGA	TGAACGGAGTTAGCTGTGTG
GAPDH	TGTGTCCGTCGTGGATCTGA	TTGCTGTTGAAGTCGCAGGAG
Primers used in vector constructed		
Coronin1-pro	GAAACAGGGTCTCACTATGTAGC	GTCAAGACAATGAATGAGCAGCG
FOXO1-up	CATCGAGAGCTCAGCCGAGAAGAG	GAATGATGGACTCCATGTCACAGTC

## Data Availability

The data that support the findings of this study are available from the corresponding author upon reasonable request.
